# Gender-based violence: Experiences from two tertiary care settings in Sri Lanka

**DOI:** 10.12688/f1000research.23120.1

**Published:** 2020-04-17

**Authors:** Dasuni Yahanika Pathiraja, Ramya Priyanwada Pathiraja, Lakshmen Senanayake, Rukshani Mayawanthi Edirisinghe, Nethanjalie Mapitigama

**Affiliations:** 1 Department of Obstetrics and Gynaecology, St. George Hospital, Kogarah, NSW, 2217, Australia; 2 Department of Obstetrics and Gynaecology, Faculty of Medical Sciences, University of Sri Jayewardenapura, Gangodawila, Nugegoda, Sri Lanka; 3 Ministry of Health, Colombo, Sri Lanka; 4 Family Health Bureau, Ministry of Health, Colombo, Sri Lanka

**Keywords:** Gender-based violence, tertiary care settings, Sri Lanka

## Abstract

**Background:** This study aimed to obtain an overview of survivors of gender-based violence GBV who seek care, different types and consequences of (GBV), their modes of referral, factors associated with GBV, characteristics of the perpetrators, health-seeking behavior of the care-seekers and the service provided by GBV Care Centers in two tertiary care settings

**Methods:** A retrospective cross-sectional study was conducted from January 2017 to December 2019 at two GBV care centers in a Women’s Hospital and a General Hospital in Colombo, Sri Lanka. Sociodemographic details of care-seekers, referral methods, types of violence experienced and their consequences, factors associated with GBV, characteristics of the perpetrator, health seeking behavior of those seeking care, and the services provided, were obtained from the hospital records.

**Results:** Records from all care seekers (n=495 women, no men) were obtained, and 488 were suitable for analysis. More women presented with GBV to the Women’s Hospital compared to the General Hospital (395 vs 93, p<0.001), and there were significant differences in modes of referral between the two hospitals. A large majority had suffered emotional and economic violence, although physical or sexual violence were the reasons for referral to the centers. Suicidal tendencies had been reported by 20%. In 94.2% of cases the husband, lover or partner was the perpetrator. Physical violence was more likely in married women, those who did not report a stable relationship, and in those who were employed. Of the 488 women, 37% were pregnant at the time of violence. Most of the women had confided with another female about the violence. Less than 5% came for follow-up.

**Conclusions: **GBV care services should be offered in all hospitals, especially those providing maternity and gynaecological care. Emotional and economic violence are common but often overlooked. There is a need to increase public awareness about GBV.

## Introduction

Gender-based violence (GBV) is one of the most notable human rights violations that occurs in most societies. Both women and men experience GBV, but the majority of survivors are women and girls ^1^. GBV could be perpetrated in multiple ways, involving sexual, physical, emotional and economical and social dimensions ^2^. GBV, including intimate partner violence (IPV) is pervasive globally and leads to significant physical and mental health problems among survivors and their children ^3^. Intimate partner violence can have an influence on the behavior of the next generation ^4^.

In the Demographic and Health Survey of Sri Lanka in 2016, a prevalence of 17% of GBV was found among ever-married women of the 15–49 age group, with a prevalence of 19.3%, 17% and 16% from the urban, estate, and rural sectors, respectively ^5^. Physical abuse during current pregnancy was reported as 4.7% in a study among 1200 women in Badulla District in 2004 ^6^. Intimate partner violence in pregnancy is known to have many negative outcomes, including miscarriages, still births and maternal deaths ^7^.

Gender and Women’s Health Unit of the Family Health Bureau of the Ministry of Health, Sri Lanka is responsible for directing the health sector response at national level. The Gender and Women’s health Unit has established dedicated GBV care centers under the name of “Mithuru Piyasa” / Natpu Nilayam, meaning the Friendly Haven, within the network of hospitals spread throughout the country to provide services to survivors of GBV. There are 70 centers spread throughout the island, following the same protocol with staff having undergone the same training. The GBV care center within the hospital is located in a space designed to maintain the privacy and confidentiality while ensuring easy and unrestricted access. The care seekers are received by a trained staff member and offered first line support, referred to as LIVES (Listen, Inquire about needs and concerns, Validate, Enhance safety, Support) ^2^, emotional support in the form of befriending, other essential services as described in the Essential Services Package ^8^ and referred to other service providers if needed. The staff of these centers undergo in-service training on a specifically designed module in order to build their capacity and skill to provide assistance, which includes active listening and emotional support (befriending) and referral to other services. The centers constantly collaborate with other service providers such as the Women and Children’s Development Unit at the Divisional Secretariats, police, probation services, Legal Aid Commission and non-governmental organization etc ^9^. Formal follow-up of survivors by staff of the care center is not promoted to ensure confidentiality and safety of the survivors and their children but voluntary follow-up visits and contacts through phone are encouraged very much.

The objectives of the current study were to obtain an overview of survivors of gender-based violence (GBV) who seek care, different types and consequences of GBV, their modes of referral, factors associated with GBV, characteristics of the perpetrators, health seeking behavior of the care seekers and the service provided by GBV Care Centers in two tertiary care settings.

## Method

### Background

A retrospective cross-sectional study of pooled data from two GBV care centers attached to two tertiary care hospitals situated in Colombo, Sri Lanka; Castle Street Hospital for Women (CSHW) and Kalubowila General Hospital (KGH), was carried out over a two-year period from January 2017 to December 2019. Both hospitals served an urban population while accepting referrals from other levels hospitals. The CSHW was an exclusive Women’s hospital.

The individual records maintained on all care seekers who had attended these two centers during the study period were perused. There were a total of 495 women (no men) seeking care for GBV from the two centers during the period of study, and all their records were available. There were seven records in which ≥5% of variables had missing values. These records were excluded resulting in 488 being selected for analysis. All the variables were directly reported by care seekers. However, three of the variables (the type of violence, the consequences of violence and reported stable relationships) needed additional interpretation by the health care professionals who entered the data into the records kept in the two GBV care centers. The professionals who worked in the GBV care centers had been trained on data collection and clear instructions had been given to them, regarding data collection and documentation of this data in the records maintained at the GBV care centers.

### Data extraction and statistical analysis

From these records, detailed information such as the sociodemographic details, referral modalities, types of violence experienced, the consequences, factors associated with GBV, the characteristics of the perpetrators, health seeking behavior of those seeking care, and the services provided including outward referrals, were extracted by trained research assistants.

In addition, safety assessment of the care seekers was carried out using a safety score as recommended by the World Health Organization (WHO) Hand Book ^2^ and highlighted in the Sri Lanka National Guideline for GBV care providers ^8^. There were five questions to assess the future safety of those seeking care, with each positive response to a question being allocated a score of one; using the cumulative scores, a risk score was calculated. The following questions were asked:

- Has the violence increased in the past year?
- Does the perpetrator consume drugs or alcohol?
- Has the perpetrator threatened to kill you?
- Does the perpetrator keep a weapon in the house?
- Are you afraid to go home?

All the records were retrieved and there was no sampling involved. Therefore, there was no selection bias. Only the records which had significant (≥5%) missing values were excluded. All the research assistants were trained for data collection and they contacted the second author (RP) for any queries and clarifications. Therefore, there was uniformity in data collection, and any bias during interpretation of data was excluded.

The extracted data was entered into data collection sheets (available as *Extended data*) ^10^, subsequently entered into an ongoing password-protected database and stored confidentially. Percentages and 95% confidence intervals (CIs) were calculated for the categorical variables. Possible associations of different factors with the types of GBV were assessed using chi square test, odds ratios (OR) and 95% CI. A p-value < 0.05 was considered as significant. Only grouped data are presented, ensuring confidentiality of the care seekers.

### Ethical approval

Approval was obtained from the Ethical Review Committee of KGH (PL/MO/2020- application no 843). Participant consent was waived because of the anonymity of the records. Permission to carry out the study was obtained from the directors of the two hospitals KGH and CSHW.

## Results

### Demographic background of patients

There were 395 (80.9%) from CSHW and 93 (19%) from KGH (p<0.001). Out of 488 women in the study, 358 (73.4%) were married. There were more married women presenting for care at CSHW compared to KGH (275/395 vs 83/93, p<0.001). There were more pregnant women presenting for care at CSHW compared to KGH (170/395 vs 10/93, p<0.001). There were more teenagers presenting for care at CSHW compared to KGH (68/395 vs 4/93, p<0.001). The proportion of women ≥40 years was larger at KGH compared to CSHW (37/93 vs 49/395, p<0.001) ( Table 1).

**Table 1.** Characteristics of women attending gender-based violence care center (n=488).

Variable		Total (N = 488)		CSHW (N = 395)		KGH (N = 93)		p-value ^a^	
		n	%	n	%	n	%	
Age in years	< 19	72	14.8	68	17.2	4	4.3	<0.001
	20–29	153	31.4	132	33.4	21	22.6	0.047
	30–39	177	36.3	146	37.0	31	33.3	0.550
	40–49	71	14.5	42	10.6	29	31.2	< 0.001
	≥ 50	15	3.0	07	1.8	08	8.6	< 0.001
Employed		169	34.6	130	32.9	39	41.9	0.115
Currently married		358	73.4	275	69.6	83	89.2	< 0.001

KGH, Kalubowila General Hospital; CSHW, Castle Street Hospital for women.

^a^Comparison between CSHW and KGH using Chi Square test.

### Referrals

A large majority (71%) who attended these two centers had been referred from the wards and outpatient department (OPD). Of the 39 (8%) women referred by the police, 38 had been referred to KGH. The police contributed to 41% of referrals to the KGH and only 0.3% of referrals to CSHW. Other sources or referral, such as general practitioners, other hospitals and lawyers, contributed to 30.4% in KGH compared to 3.0% in CSHW ( Table 2).

**Table 2.** Modes of referral to the two gender-based violence care centers (n=488).

Mode of referral	CSHW (N = 395)		KGH (N = 93)		Total	
	n	%	n	%	n	%
Referral from a ward/OPD	331	83.8	16	17.4	347	71.3
Referral from Police	01	0.3	38	41.3	39	08.0
Referral from Field Health Staff	06	1.5	02	2.2	08	1.6
Self-referral	45	11.4	08	8.7	53	10.9
Others ^a^	12	3.0	28 ^b^	30.4	40	8.2

KGH, Kalubowila General Hospital; CSHW, Castle Street Hospital for women; OPD, outpatient department.

^a^women who were referred from other hospitals, general practitioners and by lawyers etc.

^b^one response was missing

### Experiences of violence

Nearly all (94.3%) had suffered emotional violence. Economic, physical and sexual violence had been experienced by 66.6%, 64.5% and 30.3% of women, respectively, and 180 (37%) of women were pregnant at the time of violence. All four types of violence, i.e. physical, sexual, emotional and economic, were more common in those referred from the wards and OPD compared to other modes of referrals. In total, 10.8% were self-referrals and almost all of them were suffering from emotional violence. Almost all the women who had been referred by the police had experienced physical and emotional violence. All eight women referred from field health staff had emotional and economic violence ( Table 3). In cases of self-referral, most women had come to know about the GBV care center through a friend. Posters displayed at these centers also contributed to their knowledge ( Table 4). Most women had reported more than one type of violence and 14.5% had reported all four types. Of the 488 women, 69% had experienced both emotional and economic violence while 64% had experienced both physical and emotional violence.

**Table 3.** Modes of referral and types of gender-based violence (n=488).

Variable		Emotional violence n = 460 (94.3 %)	Economic violence n = 325 (66.6 %)	Physical violence n= 317 (64.5%)	Sexual violence n = 148 (30.3 %)
Referral from a ward/OPD (N = 347)	Yes	327 ^b^	244 ^b^	205	102
	No	19 ^b^	102 ^b^	142	245
Referral from Police (N = 39)	Yes	37	26 ^b^	38	13
	No	02	12 ^b^	01	26
Referral from Field Health Staff (N =08)	Yes	08	08	06	04
	No	0	0	02	04
Self-referral (N = 53)	Yes	52	32	34	17
	No	01	21	19	36
Others ^a^ (N = 41)	Yes	36 ^c^	15 ^d^	34 ^b^	12 ^e^
	No	1 ^c^	19 ^d^	06 ^b^	23 ^e^

OPD, outpatient department.

^a^Women who were referred from other hospitals, general practitioners and by lawyers etc.

^b^One response was missing.

^c^Four responses were missing.

d Seven responses were missing.

^e^six responses were missing.

**Table 4.** In case of self-referral, how the women came to know of the care centers (n=53).

How woman came to know of care center	n	Percentage	95% Confidence Interval
Through a friend	15	28	18-42
Through posters displayed in the centers	11	21	12-33
Through the media (newspapers, radio and television)	08	15	8-27
When they visited the hospital for some other health issues	07	13	7-25
Through a woman who had already attended the clinic	02	04	1-13
Others ^a^	10	19	11-31

^a^After attending a seminar, reading a book, listening to somebody else’s story.

Feeling depressed was the commonest emotional consequence and sleeping disturbances were the next commonest. Suicidal tendencies had been reported by 20%. Lack of interest in sexual relationships was the commonest sexual consequence and bruises and blackouts were the commonest physical consequences ( Table 5). In 94% of women, the husband, lover or the living in partner had been the perpetrator. However, there were women who had been subjected to GBV by both husband / lover or the living in partner as well as by other members of the family ( Table 6).

**Table 5.** Consequences following violence (n=488).

Variable	n	Percentage	95% confidence interval
**Emotional**	460	94	92-96
Depression	419	86	82-87
Sleep Disturbances	397	81	78-84
Suicidal Tendencies	97	20	17-24
Panic Attacks	67	14	11-17
**Economic**	325	67	62-71
Deprivation of money	311	64	59-68
Not recorded	14	3	2-5
**Physical**	317	65	61-69
Bruises	78	16	13-20
Blackouts	40	8	6-11
Cuts needing sutures	16	3	2-5
Burns	08	2	1-3
Dislocations	07	1	1-3
Broken bone or bones	06	1	1-3
Broken teeth	03	1	0-3
No evidence of external injuries	189	39	35-43
**Sexual**	148	30	26-35
Lack of interest in sexual relationships	111	23	19-27
Loss of libido	62	13	10-16
Dyspareunia	48	10	8-13

**Table 6.** Types of perpetrators (n=488).

Perpetrator	n	Percentage	95% Confidence Interval
Husband/Lover/Living in Partner	460	94.3	91.8-96.0
Family Member ^a^	82	16.8	13.8-20.4
Other Relations ^b^	20	4.1	2.7-6.3
Friend	05	1.0	0.4-2.4

^a^Parents, grandparents, brothers, sisters, sons, and daughters

^b^Aunts, uncles, cousins, nieces, nephews

Of the 317 women who had suffered physical violence 83% had been married (OR=4.3, 95% CI 2.8-6.6, p<0.001) and 39% had been employed (OR=1.7, 95% CI 1.1-2.6, p=0.009). Of the 148 women who had suffered sexual violence, 49% had been pregnant at the time of violence (OR=2.0, 95% CI 1.4-3.0, p <0.001). Of the 180 pregnant women with GBV, 80% had suffered economic violence (OR=2.6, 95% CI 1.7-4.1, p<0.001). Although 60% of those who had suffered sexual violence were married, marriage and reported stable relationships were associated with reduced risks of sexual violence (OR=0.4, 95% CI 0.3-0.6, p<0.001, and OR=0.5, 95% CI 0.3–0.7, p=0.001, respectively). A reported stable relationship was also associated with a reduced risk of economic violence (OR=0.2, 95% CI 0.2-0.4 p<0.001 ( Table 7).

**Table 7.** Characteristics of the women associated with the types of gender-based violence (n=488).

Variable		Emotional violence (N =460)		Economic violence (N = 325)		Physical violence (N = 317)		Sexual violence (N = 148)	
		Yes n	No n	Yes n	No n	Yes n	No n	Yes n	No n
Married (N = 358)	Yes	339 ^c^	16 ^c^	233 ^f^	119 ^f^	263 ^a^	94 ^a^	89 ^e^	264 ^e^
	No	117 ^e^	8 ^e^	91 ^e^	34 ^e^	50 ^c^	77 ^c^	58 ^e^	67 ^e^
		OR = 1.5 95% CI, 0.6-3.5 p = 0.404		OR = 0.7 95% CI, 0.5-1.1 p = 0.175		OR = 4.3 95% CI, 2.8-6.6 p < 0.001		OR = 0.4 95% CI, 0.3-0.6 p < 0.001	
Reported a stable relationship (N = 254)	Yes	240 ^b^	12 ^b^	135 ^e^	114 ^e^	163	91	60 ^c^	191 ^c^
	No	220 ^d^	10 ^d^	190 ^d^	40 ^d^	153 ^b^	79 ^b^	88 ^e^	141 ^e^
		OR = 0.9 95% CI, 0.4-2.1 p =0.828		OR = 0.2 95% CI, 0.2- 0.4 p < 0.001		OR = 0.9, 95% CI, 0.6 -1.3 p =0.682		OR = 0.5, 95% CI, 0.3-0.7 p =0.001	
Pregnant (N = 180)	Yes	173	7	144	36	112	68	72	108
	No	282 ^g^	17 ^g^	179 ^i^	118 ^i^	197 ^g^	102 ^g^	74 ^h^	224 ^h^
		OR = 1.5, 95% CI, 0.6-3.7 p = 0.383		OR = 2.6, 95% CI 1.7-4.1 p < 0.001		OR = 0.9, 95% CI, 0.6-1.3 p = 0.417		OR = 2.0, 95% CI, 1.4-3.0 p < 0.001	
Employed (N =169)	Yes	158 ^a^	10 ^a^	107 ^c^	59 ^c^	123	46	57 ^c^	109 ^c^
	No	302 ^c^	14 ^c^	218 ^d^	97 ^d^	194	125	91 ^c^	225 ^c^
		OR = 0.7 95% CI, 0.3-1.7 p = 0.464		OR = 0.8, 95% CI, 0.5-1.2 p = 0.291		OR = 1.7, 95% CI,1.1-2.6 p = 0.009		OR = 1.3, 95% CI, 0.9-1.9 p = 0.211	

CI, confidence interval; OR, odds ratio.

^a^One response was missing.

^b^Two responses were missing.

^c^Three responses were missing.

^d^Four responses were missing.

^e^Five responses were missing.

^f^Six responses were missing.

^g^Nine responses were missing.

^h^Ten responses were missing.

^i^Eleven responses were missing.

Of the 488 women 90% had spoken to someone about the violence at some point of time. In 55.1% it was either the mother/mother in law or another female family member, and it was a male family member only in 20.9%. While some had confided with a friend, very few women had spoken to a public health midwife, general practitioner or the medical officer in the outpatient department ( Table 8). On assessing the future safety of the women who had undergone GBV, the two leading risk factors were that the violence had increased during the previou *s* year (80%), and that the perpetrator consumed alcohol or drugs (60%). Of the 488 women who had undergone GBV 18.6% were afraid to go home ( Table 9). Out of a possible total of five, 19% of women had a score of ≥3 indicating a significant risk of repeat violence in the future ( Table 10).

**Table 8.** Help-seeking behavior of the women (n=488).

Variable	n	Percentage	95% confidence interval
Had spoken to someone about the violence	439	90.0	87.0-92.3
Mother/ Mother in law/Female family member	269	55.1	50.7-59.5
Male family member	102	20.9	17.5-24.7
Friend	53	10.9	48.4-13.9
Public Health Midwife	11	2.3	1.3-4.0
General Practitioner	12	2.5	1.4-4.3
Medical Officer – outpatient department	22	4.5	3.0-6.7
Clergy	01	0.2	0.04-1.2

**Table 9.** Assessment of safety (n=488).

Variable	n	Percentage	95% confidence interval
The violence has increased in the past year	390	79.9	76.1-83.2
The perpetrator consumes drugs or alcohol	293	60.0	55.6-64.3
The perpetrator has threatened to kill the woman	81	16.6	13.6-20.2
The perpetrator has a weapon in the house	21	4.3	2.8-6.5
The woman is afraid to go home	91	18.6	15.4-22.3

**Table 10.** Risk scores used to assess the future safety of the women (n=488).

Risk score	n	Percentage
0	61	12.5
1	169	35.0
2	158	32.4
3	53	10.9
4	26	5.4
5	15	3.1
Not documented	06	1.2

### Support for survivors

Nearly all women who attended these centers had received emotional support. Of the 488 women with GBV, 182 (37.3%) had been referred to services within the hospital and 64 (13%) had been referred to a psychiatrist. A total of 180 women (36.9%) were referred for services outside the hospital. These included in-depth counselling, and referral to social services, the police and for legal aid. Less than 20% of women were able to bring to the GBV care center a family member and the perpetrator for discussion and counseling respectively. Less than 5% of women voluntarily came for a follow-up visit ( Table 11). Only two women came for a second follow-up visit.

**Table 11.** Services provided at each visit to a gender-based violence care centers (n=488).

Variable	Initial visit	
	n	Percentage
Befriending/provision of emotional support	484 ^a^	99.2
Referral for other services within the hospital	182	37.3
Psychiatry	64	13.1
Medico legal	41	8.4
Medical	26	5.3
Surgical	06	1.2
Others	45	9.2
Referral for other services outside the hospital	180	36.9
Social services	45	9.2
In-depth Counseling	43	8.8
Police	42	8.6
Legal aid	38	7.8
Rehabilitation	05	1.0
Others	07	1.4
Discussion with the perpetrator	97	19.9
Discussion with family members	93	19.0

^a^Four responses were missing.

## Discussion

The policy of the GBV care centers is to provide services to both men and women survivors. However, all care seekers who attended these two centers were women. This may be because the majority of survivors of GBV are women ^11– 13^, and gender norms discourage men from disclosing GBV ^14^. Furthermore, men may not be aware of the services, and one of the centers is situated in a hospital exclusively for women. The reasons for the larger numbers presenting to the GBV care center at CSHW could be because it was better known to the public, having been established two years prior to the period of study, and also because women were more comfortable and expected better care from a center situated in a women’s hospital rather than a center situated in a general hospital. The larger number of teenagers presenting with GBV to CSHW may also be due to the same reason. The majority of care seekers being between 20–39 years probably reflect the pattern of attendees to the two hospitals. The reason for the greater proportion of GBV care seekers, who were ≥40 years of age presenting to the KGH, is unclear. Although the percentage of older women (over 50 years) in the study population was only 3%, this group should not be disregarded when planning programmes to address GBV.

Inbound referrals between the two hospitals show two different patterns. At the CSHW contributions from the wards and the OPD were high, in contrast to KGH where the major contribution had come from the Police. Differences in the operational arrangements for medicolegal services between the two hospitals, and linkages the individual centers have developed with other service providers may account for this difference. Only one in ten women being self-referrals, in spite of the fact that the centers had been operational for several years, and messages are displayed in large bill boards at the two hospitals, is of concern. The Protocol of the GBV care center indicates the need for promoting free access to survivors without formal referrals ^15^. It is also necessary to increase awareness among the public of their right to be not subjected to GBV, and also about the availability of GBV care centers.

The fact that 180 (37%) women attending the GBV care centers were pregnant indicates that the establishment of antenatal and postnatal screening should be considered, as recommended by the American College of Obstetrician and Gynaecologists 2012 ^16^. However, the large number of pregnant women in the current study is possibly due to a sample bias because 395 out of the 488 women were from a center which is situated in an exclusively women’s hospital. By contrast, in a large community based study involving 786,464 women, carried out in 2004 in the Badulla district, physical abuse was reported in only 4.7% of a current pregnancy ^6^.

The high emotional impact of GBV was evident, with 93% having had depression and 20% having had suicidal ideations. The proportion having suicidal ideation was higher among women who attended these centers relative to an earlier study carried out in 2007 in an agricultural community in the south-western region of Sri Lanka where domestic violence and abuse were seen as a cause of self-harm by ingestion of pesticide in 12% of the cases ^17^.

It is important to identify women undergoing GBV and to provide effective emotional support in order to ensure their emotional wellbeing and prevent suicides.

The fact that a high risk of GBV was associated with women who were married, those who did not have stable relationships, those who were pregnant and in those who were employed, and the husband, lover or living in partner was the commonest perpetrator in both present and past episodes, indicates the vital importance of providing family counselling services to the community at large. It is important to note that 60% of perpetrators had consumed alcohol or drugs. This aspect too can be addressed by a family counselor. In the current study, however, less than 20% of women had made use of the opportunity of obtaining these counselling services from the two centers and very few women had returned to the care center for follow up.

Help-seeking by the women was mainly focused on friends and relations. This may be due to a reluctance to divulge to an outsider the fact that they had been victims of GBV. Although very few women had sought assistance from health care providers in the community, public health midwives, general practitioners and medical officers in the community are very good sources of assistance and care to survivors of GBV. The public should be educated about this.

The main strength of this study is its large sample size (n=488) and the very high data retrieval rate (95%). However, the data were collected from GBV care centers, situated in two teaching hospitals, one of which was a hospital for women and both hospitals are situated in urban areas. Furthermore, the effects of racial/ethnic differences on GBV were not studied. Therefore, the generalizability of these findings to the community at large is limited. Nevertheless, this study gives some valuable insights to the problem of GBV in two urban areas of Sri Lanka.

## Conclusions

Establishment of dedicated GBV care centers within hospitals, especially those with maternity and gynaecology services, could promote identification of GBV survivors, and delivery of essential services to them. It is important to recognize the gravity of the emotional impact on survivors of GBV and provide effective and adequate emotional support to assist them and prevent suicide, when managing these survivors, especially if they are pregnant. GBV care centers need to be supported through a network of professionals skilled in family counseling to support the efforts made by the staff to help in conflicting resolution. Effective public awareness programmes on the availability of Gender Based Violence care centers dedicated to survivors of GBV and also the services provided by these centers should be conducted to ensure optimal utilization of these centers and provide care to survivors of GBV who often suffer at home in silence.

## Data availability

### Underlying data

These data are available from the Medical Directors of the KGH and CSHW, which are governed by the Ministry of Health, Sri Lanka. Restrictions apply to the availability of these data and are not publicly available. Readers or reviewers who wish to access these data and also those who are interested to use these data for future research can contact Gender and Women’s Health unit, Family Health Bureau ( info@fhb.health.gov.lk), Ministry of Health, Sri Lanka.

Research data were stored in a tabular format, in SPSS (Statistical Package for the Social Sciences, Version 25). Numbers 1–93 and 94–488, contained data from the GBV care centers at KGH and CSHW respectively. Each row represents data from an individual woman and each column to represents a variable from the data sheet. There were 141 variables in the data sheet.

### Extended data

Figshare: Gender-based violence: Experiences from two tertiary care settings in Sri Lanka. https://doi.org/10.6084/m9.figshare.12084219.v1 ^10^.

This project contains the data extraction sheets used in the present study.

Extended data are available under the terms of the Creative Commons Attribution 4.0 International license (CC-BY 4.0).
