# Supplementary material for: Gender-based violence: Experiences from two tertiary care settings in Sri Lanka
Source: F1000Res. 2020 Apr 17;9:269. [Version 1] doi: 10.12688/f1000research.23120.1 (PMC7199284; doi:10.12688/f1000research.23120.1)
Supplement: Peer review history

# Peer review: Reviewer response for version 1

**DOI:** 10.5256/f1000research.25523.r62513

**Published:** 2020-05-11

**Authors:** Nalika Gunawardena

**Affiliations:** 1 Country Office for Sri Lanka, World Health Organisation, Colombo, Sri Lanka

A well designed study on an important topic.

 The correct title of the one of the hospital settings is Colombo South Teaching Hospital and not Kalubowila General Hospital (KGH). The authors need to attend to the correction. It would have been worthwhile if the researchers could have substantiated the findings with a few qualitative inquiries among the users. The conclusions and the recommendations are relevant to improve the services.

Is the work clearly and accurately presented and does it cite the current literature?

Yes

If applicable, is the statistical analysis and its interpretation appropriate?

Yes

Are all the source data underlying the results available to ensure full reproducibility?

Yes

Is the study design appropriate and is the work technically sound?

Yes

Are the conclusions drawn adequately supported by the results?

Yes

Are sufficient details of methods and analysis provided to allow replication by others?

Yes

Reviewer Expertise:

NA

I confirm that I have read this submission and believe that I have an appropriate level of expertise to confirm that it is of an acceptable scientific standard.

# Peer review: Reviewer response for version 1

**DOI:** 10.5256/f1000research.25523.r62512

**Published:** 2020-05-04

**Authors:** Achini C. Jayatilleke

**Affiliations:** 1 Post Graduate Institute of Medicine, University of Colombo, Colombo, Sri Lanka

This study provides an overview of GBV survivors presented to two urban GBV care services.

 The article is well written, and carries important findings that will help improve GBV service provision in Sri Lankan hospitals.

 The study explains the types of survivors seeking care, the different types of violence they were experiencing, the health effects of such violence, health seeking behaviors of survivors, etc. 

 According to the study, a large majority had suffered emotional and economic violence, although physical or sexual violence were the reasons for referral to the centers. This finding needs to be highlighted and could be useful for future policy decisions on GBV referrals. The alarming number of suicidal tendencies indicate the need of their urgent psycho-social support along with follow up. However, it is disturbing to note that only 5% came for follow-ups, and only 2 attended a second follow up. This point needs discussion and recommendations should be made to improve GBV follow ups in the GBV care centers.

Is the work clearly and accurately presented and does it cite the current literature?

Yes

If applicable, is the statistical analysis and its interpretation appropriate?

Yes

Are all the source data underlying the results available to ensure full reproducibility?

Yes

Is the study design appropriate and is the work technically sound?

Yes

Are the conclusions drawn adequately supported by the results?

Yes

Are sufficient details of methods and analysis provided to allow replication by others?

Yes

Reviewer Expertise:

Gender Based Violence

I confirm that I have read this submission and believe that I have an appropriate level of expertise to confirm that it is of an acceptable scientific standard.

# Peer review: Reviewer response for version 1

**DOI:** 10.5256/f1000research.25523.r62514

**Published:** 2020-04-30

**Authors:** Vathsala Illesinghe

**Affiliations:** 1 Ryerson University, Toronto, ON, Canada

This paper presents findings of a **desk review** or secondary analysis of data routinely collected at two health care institutions in Sri Lanka from those who seek services for GBV (intimate partner violence predominantly) during a specified time period. 

 While this type of study can provide a descriptive summary of the characteristics of those who seek care and the nature of services that could be provided to them, it will not achieve all of the objectives nor support all of the conclusions stated in the paper. For example, the mentioned objective(s) of "obtain(ing) an overview of [...] factors associated with GBV" would not be possible without an analytical component/comparison group who did not experience GBV as opposed to those who did not report specific types of GBV (as in table 7). 

 The following aspects of this paper could be improved to increase the relevance and useful of this publication. The results section of this paper will benefit from a revision (see details below).

**Introduction**: 

- The focus of the study is not clear whether it is Gender Based Violence (GBV), GBV against women: most GBV in Sri Lanka is male violence against women in the context of marital or intimate relationships. 
- Justify the choice of study settings: These two hospitals represent diverse health care institutions, one being a maternity hospital and a referral center for the rest of the country requires a justification as to why these two hospitals were chosen; it is relevant for the interpretation of results. 

**Methods:**

 Ethics approval: Desk reviews would not require a statement about individual consent. Whether protocols/pre-approvals are in place to use routinely collected data for research purposes could be mentioned. How was anonymity of data assured? Were there any issues of data inconsistency in the two settings? 

 Some of the questions used to assess safety were different from the WHO questions:

- Has the physical violence happened more often or gotten worse over the past 6 months?
- Has he ever used a weapon or threatened you with a weapon?
- Has he ever tried to strangle you?
- Do you believe he could kill you?
- Has he ever beaten you when you were pregnant?
- Is he violently and constantly jealous of you?

What is the rationale for the use of different questions? The questions used by the authors could be more appropriate to the study setting; this could be justified using literature and other evidence. 

**Results**:

- The statement 'there were 395 (80.9%) from CSHW and 93 (19%) from KGH ( **p<0.001)**' is not meaningful without a baseline for comparison. Are you saying that there is a higher proportion of women who reported IPV among all the women/patients who were admitted to the hospital in CSHW compared to the KGH? If so, could this be because CSHW is a women's hospital and only admits women compared to KGH which admits both men and women? A proportion of 395/total cases collected would not be a meaningful comparison. 
- Interpretation of the results should be reviewed throughout. Rather than "there were more teenagers presenting for care at CSHW compared to KGH (68/395 vs 4/93, p<0.001)' it is better stated as 'a higher proportion of those who presented to CSHW were aged <19 years compared to the proportion of women who were of that age group presenting to KGH. 
- Table 7. Characteristics of the women associated with the types of gender-based violence is hard to interpret without proportions. Interpretation is not clear 'of the 317 women who had suffered physical violence 83% had been married (OR=4.3, 95% CI 2.8-6.6, p<0.001)' as opposed to what? (those who did not suffer physical violence - but could they have suffered other types of violence? Which types?) 
- Tables 8 and 9, the calculation/interpretation of the confidence intervals are not clear. 

**Discussion:**

- The authors have addressed some of the biases and discussed salient points of this study. The results of a care center could not be compared to community based samples and prevalence. 
- This could be an error -  "a large community based study involving **786,464 **women," - the sample size of this study was much smaller. 
- The authors must also pay attention to making conclusions about multiple variables without doing multivariate analysis - for example, it cannot be concluded that  - "a high risk of GBV was associated with women who were married, those who did not have stable relationships, those who were pregnant and in those who were employed, and the husband, lover or living in partner was the commonest perpetrator in both present and past episodes [...] because these variables were not compared with each other. 
- Findings about help seeking, it must be noted, is based on the experiences of a sample that presented to hospital, and as such, cannot be generalized to those who have not done so. Hence it cannot give insights in to the "problem of GBV in two urban areas of Sri Lanka".

**Conclusions**:

- The findings point to the nature of services needed and gaps in those provided to women who present to two large hospitals in Sri Lanka. For example, the findings can support the need for psycho-social services for survivors and the requirements for safety planning and interventions. Any other extrapolation to the community level would not be supported by the data collected and presented here. 

Is the work clearly and accurately presented and does it cite the current literature?

Partly

If applicable, is the statistical analysis and its interpretation appropriate?

Partly

Are all the source data underlying the results available to ensure full reproducibility?

Partly

Is the study design appropriate and is the work technically sound?

Partly

Are the conclusions drawn adequately supported by the results?

Partly

Are sufficient details of methods and analysis provided to allow replication by others?

Partly

Reviewer Expertise:

Violence against women

I confirm that I have read this submission and believe that I have an appropriate level of expertise to confirm that it is of an acceptable scientific standard, however I have significant reservations, as outlined above.

# Peer review: Reviewer response for version 1

**DOI:** 10.5256/f1000research.25523.r62515

**Published:** 2020-04-27

**Authors:** Sir Sabaratnam Arulkumaran

**Affiliations:** 1 St George's, University of London, London, UK

The article “Gender-based violence: Experience from two tertiary care settings in Sri Lanka” is based on the retrospective analysis of well recorded data of 488 women who attended special clinics in one women’s hospital and one general hospital in the capital city of Colombo, Sri Lanka. The analysis consists of socio-demographic factors, details of care seekers, how they ended in the clinic and the types of violence i.e. economic, emotional, physical or sexual. The tendency for suicide was notable with 20% respondents admitting that they had encountered these thoughts. In 92% of cases, the husband, partner or lover were the perpetrators. Married women and those not in a stable relationship experienced more physical violence. Emotional and economic violence were the commonest form of GBV.

 This study highlights the important issues of economic and emotional GBV. This may be linked to the fact that these women may be no or low wage earners. The low follow up visit rates indicates that either the women are busy or they feel that the advice/services they receive may not be adequate. The conclusion suggests that there is a need to increase public awareness. One wonders whether this could be achieved by incorporating the GBV issue in schools and religious events. This paper is an important contribution to highlight the problem. Future studies are needed to focus on effective interventions and outcomes.

 The work is clearly and adequately presented with citation to recent references. It is a detailed analysis of well recorded retrospective data. Sufficient details on methods and analysis is provided for others to produce similar work. I am not a qualified statistician to verify the accuracy of the analysis. Availability of the source data and how to access the data is provided by the authors. Conclusions are supported by the data and analysis provided.

Is the work clearly and accurately presented and does it cite the current literature?

Yes

If applicable, is the statistical analysis and its interpretation appropriate?

I cannot comment. A qualified statistician is required.

Are all the source data underlying the results available to ensure full reproducibility?

Yes

Is the study design appropriate and is the work technically sound?

Yes

Are the conclusions drawn adequately supported by the results?

Yes

Are sufficient details of methods and analysis provided to allow replication by others?

Yes

Reviewer Expertise:

Obstetrics and Women’s Health

I confirm that I have read this submission and believe that I have an appropriate level of expertise to confirm that it is of an acceptable scientific standard.
